# Effect of mammography screening on the long-term survival of breast cancer patients: results from the National Cancer Screening Program in Korea

**DOI:** 10.4178/epih.e2022094

**Published:** 2022-10-26

**Authors:** Xuan Quy Luu, Kyeongmin Lee, Jae Kwan Jun, Mina Suh, Kyu-Won Jung, Kui Son Choi

**Affiliations:** 1 Department of Cancer Control and Population Health, Graduate School of Cancer Science and Policy, National Cancer Center, Goyang, Korea; 2 National Cancer Control Institute, National Cancer Center, Goyang, Korea

**Keywords:** Mass screening, Breast neoplasms, Mammography, Korea

## Abstract

**OBJECTIVES:**

This study investigated the effect of mammography screening on the long-term survival of breast cancer (BC) patients aged 40 years or older according to their screening history and duration since screening.

**METHODS:**

The study cohort was organized from 3 nationwide databases of the Korean National Cancer Screening Program, the Korean Central Cancer Registry, and death certificates. We included 24,387 women diagnosed with invasive BC or ductal carcinoma in situ in 2008 and 2009 and followed up until December 31, 2019. Cox proportional-hazards regression was used to investigate the effect of BC screening on the risk of death.

**RESULTS:**

Overall, 20,916 of 24,387 patients (85.8%) were alive at the end of the follow-up period (median: 10.5 years). The long-term survival rate was significantly lower in the never-screened group (80.3%) than in the screened group (88.9%) (p<0.001). A 35% reduction in the risk of BC death (hazard ratio [HR], 0.65; 95% confidence interval [CI], 0.60 to 0.70) from screening was observed. A subgroup analysis according to the cancer stage showed 62%, 36%, and 24% lower risks of BC death for the localized stage, regional stage, and distant stage, respectively. Women aged 40–49 years received the least benefit from BC screening (HR, 0.71; 95% CI, 0.62 to 0.81).

**CONCLUSIONS:**

Mammography screening was effective in reducing the risk of BC-specific death in Asian women across all cancer stages. However, this effect was relatively small among women in their 40s, suggesting that more detailed and specialized screening strategies are needed for that age group.

## GRAPHICAL ABSTRACT

[Fig f2-epih-44-e2022094]

## INTRODUCTION

Breast cancer (BC) is the most commonly diagnosed cancer type globally in terms of both the number of new cases and the age-standardized incidence. In 2020, BC accounted for 11.7% of new cancer cases [1]. However, the distribution of BC cases is skewed; approximately more than 80% of BC cases occur in countries with high or very high human development indices. To reduce the high burden of BC, several countries have adopted national cancer screening programs [2], which mostly use mammography as the main screening modality. With the geo-ethnic differences in BC epidemiology, the challenges in implementing the mammography screening program vary across regions [3–5]. In Asia, especially, BC screening has several additional challenges, including screening women in their 40s and the accuracy of mammography in populations with a relatively high prevalence of dense breasts and the relationship thereof to overdiagnosis [3,6]. Therefore, BC screening in Asia should be considered carefully with region-specific evidence [3,4]. However, studies investigating the effectiveness of mammography screening programs in Asia are still limited [7–9].

In Korea, BC is one of the most common cancer types in women. In contrast with the decreasing overall cancer incidence, BC incidence is steadily increasing. In 2018, the age-standardized rate of BC (57.9 per 100,000) was almost 3 times higher than those of colorectal cancer (20.6 per 100,000) and stomach cancer (18.3 per 100,000) in women [10]. BC incidence peaked at 45–49 years before gradually declining with age [10]. Currently, the Korean National Cancer Screening Program (KNCSP) provides biannual mammography screening examinations for all women aged 40 years or older based on the age-specific pattern of BC incidence in Korea [11]. A previous study evaluated the effect of mammography on BC mortality [12], but it was limited in assessing patients’ long-term survival and the time interval since screening exposure.

Therefore, this study investigated the effect of mammography on the long-term survival of BC patients aged 40 years or older according to the screening history and time since screening using the KNCSP database. We also assessed the association between mammography screening and survival for ductal carcinoma in situ (DCIS) cases.

## MATERIALS AND METHODS

### Study materials

The baseline population comprised 24,454 patients aged 40–79 years who were diagnosed with BC or DCIS between January 1, 2008, and December 31, 2009, and registered in the Korea Central Cancer Registry (KCCR), which covered more than 95% of cancer cases in Korea [10]. Subsequently, we excluded 67 BC patients identified from only death certificates without diagnostic information. Finally, a total of 24,387 women were included in our final analysis.

Using each resident’s unique 13-digit registration number, we linked our baseline BC patients identified from the KCCR and the KNCSP database for information on the history of mammographic screening between 2002 and 2009. We followed them up from the date of BC diagnosis until the end of 2019, allowing us to determine the long-term survival of the entire study population for at least 10 years. The date and cause of death were ascertained from the death certificates provided by Statistics Korea.

### Measurements

Information on the date of the primary diagnosis and tumor characteristics was obtained from the KCCR database [10]. We used the International Classification of Diseases, 10th revision (ICD-10) codes [13], and the BC cases were defined as those with the ICD-10 codes C50 and D05. The anatomic sites were grouped into the inner part, the outer part, central portion, and others (nipple and axillary tail of the breast) using the topographical code of the International Classification of Diseases for Oncology, third edition (ICD-O-3) [14]. For the histological subtype, we divided the patients into 4 main groups based on ICD-O-3 morphology codes, as suggested by a previous study: DCIS, ductal carcinoma, lobular carcinoma, and others [15]. The stage at diagnosis was presented as DCIS, localized stage, regional stage, or distant stage according to the summary stage classification in the Surveillance, Epidemiology, and End Results (SEER) Cancer Statistics Review of the National Cancer Institute [16].

The mammography screening history and the basic socio-demographic information were extracted from the KNCSP database. The mammography screening exposure of BC patients was assessed using screening records from 2002 to 2009, when the KNCSP for BC was introduced [11]. Socioeconomic status was categorized into 3 groups according to the health insurance type: Medical Aid program recipients (people who live under the poverty line and receive livelihood assistance from the government), National Health Insurance beneficiaries with a premium of the 50th percentile or lower, and National Health Insurance beneficiaries with a premium above the 50th percentile.

### Study outcome

The primary outcome of the study was the long-term survival of BC patients according to their mammography screening history. To ascertain death, all BC patients were followed for at least 10 years or more. The primary outcome of this study was BC-specific mortality. All-cause deaths, including and excluding those from BC as secondary outcomes, were also assessed to adjust for methodological bias, such as misclassification bias and competing risks. Furthermore, the number of person-years of all BC patients was measured from the date of BC diagnosis to the date of death or the end of follow-up, whichever occurred first, corresponding to the end of the observation period (December 31, 2019).

### Statistical analysis

The baseline characteristics, including socio-demographic information and tumor characteristics, of the screened and never-screened patients were compared using the chi-square test. Kaplan–Meier analysis and the log-rank test were used to compare the survival of subgroups according to screening history.

We conducted Cox proportional-hazards regression analysis to estimate the hazard ratios (HRs) with 95% confidence intervals (CIs) for investigating the effect of BC screening on the risk of BC death. We assessed the HRs for all-cause death, and non-BC death, to adjust for methodological bias, such as competing risks. All models were adjusted for age, socioeconomic status, and tumor characteristics such as cancer stage, anatomic site, and histological subtype. Additionally, the models were run on several subpopulations stratified by age and cancer stage to investigate variation in the BC screening effects in different groups.

The net benefit of mammography screening was estimated to address the risk of self-selection bias in a cohort study using a formula suggested by a previous study: net benefit=(HR^b^−HR^a^)/HR^b^×100, where HR^b^ represents the HR for total mortality except BC, and HR^a^ represents the HR for BC-specific mortality [17]. SAS version 9.4 (SAS Institute Inc., Cary, NC, USA) was used for all the statistical analyses, and p-values of <0.05 denoted statistical significance.

### Ethics statement

The current retrospective study was approved by the Institutional Review Board of the National Cancer Center, Korea (No. NCCNCS08129). We used de-identified data of a relatively large population from the National Health Insurance Service database, and the requirement for informed consent was waived.

## RESULTS

**Figure 1:**
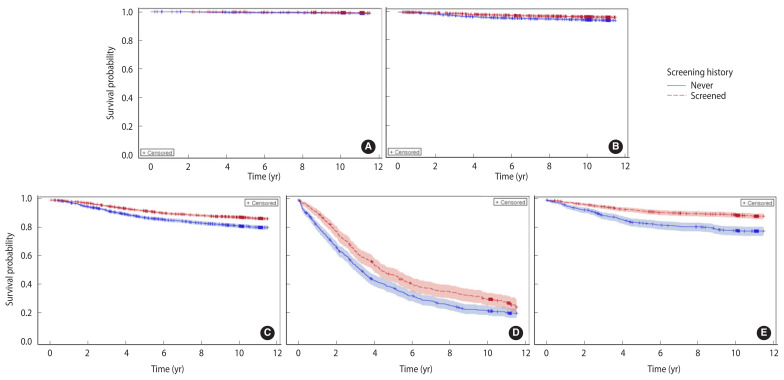
Product-limit breast cancer-specific survival estimates according to screening history-based SEER stages (A) DCIS, (B) localized, (C) regional, (D) distant, and (E) unknown. SEER, Surveillance, Epidemiology, and End Results; DCIS, ductal carcinoma in situ.

The baseline characteristics of the BC patients are presented in Table 1. Of the 24,387 BC patients, 8,823 (36.2%) were never screened and 15,564 (63.8%) were screened for BC. Significant differences were found in socio-demographic and tumor characteristics between the never-screened and screened patients. BC involving the inner and outer parts of the breast, DCIS, and localized BC were significantly more prevalent among the screened patients than among the never-screened patients (p<0.001). Of the screened patients, 54.6% underwent screening only once, and 68.5% underwent screening within a year of the date of cancer diagnosis. Overall, 3,471 patients died (14.2%), of whom 2,614 died from BC during the median follow-up duration of 10.5 years (interquartile range, 10.3–11.5). The survival rate was significantly higher among the screened patients (88.9%) than among the never-screened patients (80.3%) (p<0.001). Similar trends of survival were also observed in each cancer stage except DCIS (p=0.28, Figure 1A). The long-term survival was significantly different according to sociodemographic characteristics and screening histories (Supplementary Material 1). The risk of BC-specific death (HRs) increased significantly with age, lower socioeconomic status, and advanced stage at diagnosis.

The HRs for all-cause death, BC-specific death, and non-BC death according to the screening history are shown in Table 2. For model 1, after adjusting for age and economic status, mammography screening was associated with an approximately 53% lower risk of death from BC (HR, 0.47; 95% CI, 0.44 to 0.51). This figure was 35% for model 2, with additional adjustment of tumor characteristics (HR, 0.65; 95% CI, 0.60 to 70) and the fully adjusted model 3 with invasive cancer only (HR, 0.65; 95% CI, 0.60 to 0.70). A similar decrease was observed for overall death and death from conditions other than BC, with fully adjusted HRs (including invasive cancer only) of 0.68 (95% CI, 0.63 to 0.72) and 0.74 (95% CI, 0.64 to 0.86), respectively.

The effect of screening on BC mortality varied with age and the stage of BC at diagnosis (Table 3). The risk reduction for BC-specific death was the highest for patients older than 70 years (HR, 0.50; 95% CI, 0.40 to 0.63) who had been screened and the lowest for those in their 40s (HR, 0.71; 95% CI, 0.62 to 0.81). The largest risk reduction for BC-specific death was observed in patients with localized BC who had been screened (HR, 0.58; 95% CI, 0.49 to 0.70). For cases of distant-stage cancer, the risk of death was significantly reduced by approximately 24% (HR, 0.76; 95% CI, 0.66 to 0.89). However, there was no significant difference in BC-specific survival among patients diagnosed with DCIS (HR, 0.62; 95% CI, 0.26 to 1.43; p=0.280). Overall, mammography screening was significantly associated with 42%, 36%, and 24% lower risks of BC death in the localized stage, regional stage, and distant stage subgroups, respectively (Table 3). Given the similar results for all-cause death, BC-specific death, and other causes of death except BC, further subgroup analyses were conducted (Supplementary Material 2).

Additionally, our results showed similar effects of screening on BC death between people with time intervals since the last screening of less than 1 year (HR, 0.63; 95% CI, 0.57 to 0.69) and 1–2 years (HR, 0.65; 95% CI, 0.56 to 0.76) (Table 4). A 25% lower risk of death from BC was still observed among people who had their last screening more than 2 years earlier (2–3 years: HR, 0.75; 95% CI, 0.62 to 0.91; >3 years: HR, 0.75; 95% CI, 0.62 to 0.91). Furthermore, women who underwent mammography screening more than 3 times before BC diagnosis had a statistically significant 48% reduction in the risk of death from BC.

## DISCUSSION

Long-term follow-up studies estimating the screening effects of BC patients in Asian women are very limited. The current study reported that the rate of BC-specific deaths was 35% lower among screened BC patients relative to their never-screened counterparts (HR, 0.65; 95% CI, 0.60 to 0.70) based on 10-year follow-up data. This study also provided additional evidence on the stage-specific screening effect; the reductions in the risk of BC death after screening were 42%, 36%, and 26% for patients diagnosed with localized, regional, and distant stages, respectively. A study conducted in Finland involving participants predominantly aged ≥40 years reported that patients with BC detected in a screening program had a 41% lower risk of BC death than those with BC detected outside the screening program after 15 years of follow-up [18]. Similarly, a large-scale study using data from the Swedish Cancer Registry highlighted that 40-year-old to 69-year-old women who underwent mammography had a 41% lower risk of BC death within 10 years [19]. A 57% BC mortality rate reduction in women aged 50–69 years was reported by Kaplan et al. [20] for 5-year survival, and a further reduction was expected at 10 years as the effect of lead-time bias. Kalager et al. [21] reported a 14% reduction in the BC mortality rate after the introduction of a screening program relative to that before the program, which is lower than that reported by our study and other mentioned studies [18,19]. The main reason for this difference is that Kalager et al. [21] divided BC patients into 2 groups (pre-program and post-program) for the analysis, without considering their participation status. Therefore, the effect of mammography on survival may have been underestimated, as some patients in the post-program group did not participate in the screening program.

The improvement in the overall survival of screened patients could be explained by the stage-shift effect of screening. Screened people have a higher likelihood of cancer being detected at an early or even pre-cancerous stage with a better prognosis, which is well documented in previous studies on mammography screening [7,12,19,21–23] and in our study results. Furthermore, as the basic mechanism of screening is to detect cancer early, even within a stage, screened people have a higher likelihood of cancer being detected at the beginning of this stage. Thus, the early initiation of treatment could have a favorable effect on patient outcomes. Furthermore, the prognosis of patients is also related to the histological characteristics of the tumor. Screening-detected cancer cases are likely to be slowly progressing cancers, which also have a better prognosis than same-stage tumors with more aggressive behavior [24]. Although there is limited research on whether mammography screening improves stage-matched survival, better survival was observed in mammography-examined women among patients with similar tumor sizes, showing consistent results with our findings. Results from a mammographic screening study indicated that improved survival among screened people existed across all subgroup analyses according to tumor size (1–10, 11–20, and 21–50 mm) [18]. Similarly, Joensuu et al. [25] reported an improvement in distant disease-free survival among screened individuals in all tumor size strata, even for patients with large tumors (30 mm or more).

Mammography screening among women younger than 50 years is controversial, as limited evidence supports its cost-effectiveness among this age group [4,5]. Notwithstanding, some Asian countries have included women in their 40s in their screening guidelines, given the different epidemiological characteristics of Asian populations [3–5]. In the current study, we found that the youngest age group (40–49 years) received the least benefit from mammography screening related to long-term survival (HR, 0.71; 95% CI, 0.62 to 0.81). The higher prevalence of dense breasts in younger women had been addressed by a Korean study; whereas the proportion of women with dense breasts was approximately more than 80% among women in their 40s attending mammography screening, this proportion decreased in the older age groups [26]. The accuracy of mammography screening may be lower for this age group [4]. Therefore, screening for women in their 40s should incorporate age-specific strategies including an appropriate modality and screening interval [4,27–29]. The U.S. Preventive Services Task Force recommendation indicates that screening at the age of 40 years should be based on the individual risk and benefits of mammography [27]. According to the recommendations of the American Cancer Society, 40-year to 44-year old women can undergo mammography screening every year; however, women aged 45–54 and ≥55 years should screen annually and biennially, respectively [28]. The American College of Radiology also recommends annual mammography screening for women starting from 40 years old [29].

Overdiagnosis by BC screening refers to cases that are detected and diagnosed as BC but never progress to symptomatic and aggressive cancer cases. Therefore, the detection of these cases does not contribute to reducing death and causes harm to patients, such as psychological consequences or unnecessary treatments [30,31]. In the current study, we found a significantly higher proportion of DCIS cases among the screened group (13.9%) than among the never-screened group (10.0%) but no significant reduction of the long-term BC mortality rate attributable to screening. While this might be associated with the successful treatment of early cancer, there could also be several cases of overdiagnosis in this group. It is well known that DCIS patients have an excellent prognosis, with a survival rate of more than 95% during the long-term follow-up [31], which is consistent with the long-term survival rate of 96.4% in our study. Moreover, a study from SEER indicated that low-grade DCIS patients in surgery and non-surgery groups had the same survival rates [32]. In contrast, women diagnosed with DCIS are not well aware of their actual risk of developing invasive cancer, which worsens their screening-related psychological issues [31]. Given the increasing trend of DCIS associated with the widespread implementation of screening, it is essential to improve the understanding of DCIS in the population and a shared decision-making strategy for DCIS treatment between patients and physicians to minimize the risk of overdiagnosis and overtreatment.

Mammography screening has challenges that should be taken into careful consideration when evaluating its effectiveness. Firstly, the cancer cases detected by screening appear to have demonstrated longer survival, which is attributable to the earlier diagnosis (i.e., lead-time bias). In our cohort, the effect of lead-time bias may have been minimal due to the follow-up duration of at least 10 years for all patients. In addition, there is also the risk of selection bias stemming from the different baseline characteristics of people who participate in screening and those who do not. To partially control this in our study, we used several models with different population levels of adjustment of socioeconomic and tumor characteristics. Since BC screening is universally provided and the lifetime screening rate is more than 80% [11], the selection bias would be small. Furthermore, we also used a formula suggested by a previous study to address self-selection bias in a cohort study [17], and the calculated net benefit from mammography screening in our study was 12.2% for invasive cancer only (Table 2) and 17.6% after excluding DCIS and distant cancer cases (Supplementary Material 3).

Nevertheless, our study had several limitations. First, our study could not combine opportunistic screening information with the screening history. People who did not receive screening through the KNCSP may have already undergone opportunistic screening, which is often performed using ultrasonography as a screening test. Therefore, the effect of BC screening through the KNCSP could be underestimated. Second, our study only assessed the extent of BC based on the summary stage classification by the SEER Cancer Statistics Review of the National Cancer Institute [16], which had limitations in investigating the stage-specific effect. Lastly, as our study covered all BC patients diagnosed in 2008 and 2009 in Korea, it was not possible to include information about treatment, which is a strong predictor of patient prognosis, in our analysis. However, we believe that the treatments received by people in the screened and never-screened groups would have been similar at the same stage, as all Korean residents are enrolled in the National Health Insurance Service. Despite these limitations, this is one of the first studies evaluating the effect of mammography on the long-term survival of BC patients in Asia, to the best of our knowledge. Our study linked information at the individual level from 3 national databases with nearly complete data on screening, cancer, and death information. This makes our findings relatively generalizable to the entire population of the country.

In conclusion, our study found a significant improvement in the survival of BC patients after mammography screening during a long-term follow-up of 10 years. Our results also indicated a smaller effect of mammography among women in their 40s, who require more detailed and specialized screening strategies. Future studies should have appropriate designs to directly address the overdiagnosis by mammography screening, especially among DCIS patients.

## Figures and Tables

**Figure f2-epih-44-e2022094:**
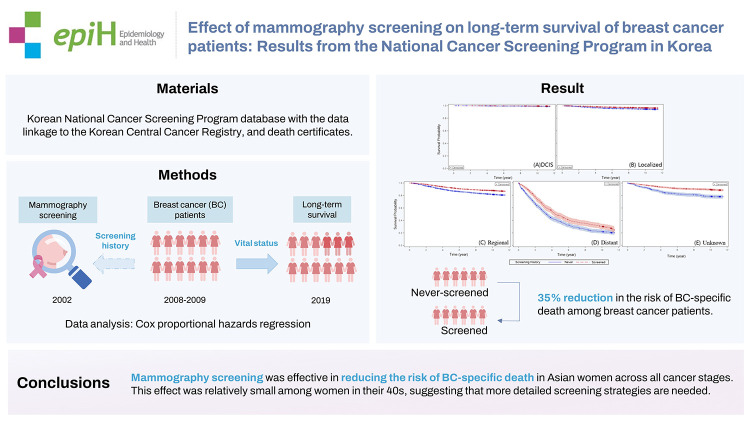


**Table 1:** Baseline characteristics of BC patients diagnosed from 2008 to 2009

Characteristics	Total (n=24,387)	Never-screened (n=8,823)	Screened (n=15,564)	p-value
Age at diagnosis (yr)				<0.001
40–49	10,387 (42.6)	4,176 (47.3)	6,211 (39.9)	
50–59	8,409 (34.5)	2,739 (31.0)	5,670 (36.4)	
60–69	3,909 (16.0)	1,171 (13.3)	2,738 (17.6)	
70–79	1,682 (6.9)	737 (8.4)	945 (6.1)	

Socioeconomic status				<0.001
NHI premium, upper 50%	12,361 (50.7)	4,539 (51.4)	7,822 (50.3)	
NHI premium, lower 50%	11,033 (45.2)	3,917 (44.4)	7,116 (45.7)	
MAP	993 (4.1)	367 (4.2)	626 (4.0)	

Anatomical site				<0.001
Inner part	3,833 (15.7)	1,330 (15.1)	2,503 (16.1)	
Outer part	9,295 (38.1)	3,175 (36.0)	6,120 (39.3)	
Central portion	1,127 (4.6)	421 (4.8)	706 (4.5)	
Others	10,132 (41.5)	3,897 (44.2)	6,235 (40.1)	

Stage at diagnosis				<0.001
DCIS	3,046 (12.5)	883 (10.0)	2,163 (13.9)	
Localized	11,374 (46.6)	3,829 (43.4)	7,545 (48.5)	
Regional	7,232 (29.7)	2,846 (32.3)	4,386 (28.2)	
Distant	1,034 (4.2)	612 (6.9)	422 (2.7)	
Unknown	1,701 (7.0)	653 (7.4)	1,048 (6.7)	

Histological subtype				<0.001
DICS	3,046 (12.5)	883 (10.0)	2,163 (13.9)	
Ductal carcinoma	18,432 (75.6)	6,789 (76.9)	11,643 (74.8)	
Lobular carcinoma	1,158 (4.7)	431 (4.9)	727 (4.7)	
Others	1,751 (7.2)	720 (8.2)	1,031 (6.6)	

Screening frequency (times)				NA
Never	8,823 (36.2)	8,823 (100)	-	
1	8,505 (34.9)	-	8,505 (54.6)	
2	4,490 (18.4)	-	4,490 (28.8)	
≥3	2,569 (10.5)	-	2,569 (16.5)	

Time interval since screening (yr)				NA
Never	8,823 (36.2)	8,823 (100)	-	
>3	1,231 (5.0)	-	1,231 (7.9)	
2–3	1,120 (4.6)	-	1,120 (7.2)	
1–2	2,548 (10.4)	-	2,548 (16.4)	
<1	10,665 (43.7)	-	10,665 (68.5)	

Survival status				<0.001
Alive	20,916 (85.8)	7,081 (80.3)	13,835 (88.9)	
BC death	2,614 (10.7)	1,371 (15.5)	1,243 (8.0)	
Non-BC death	857 (3.5)	371 (4.2)	486 (3.1)	

Values are presented as number (%).

BC, breast cancer; NHI, National Health Insurance; MAP, Medical Aid Program; DCIS, ductal carcinoma in situ; NA, not available.

**Table 2:** Hazard ratios for different causes of death according to the screening history

Variables	Death	Person-years	Death rate per 1,000	Crude	Model 1^1^	Model 2^2^	Model 3^3^
All-cause death							
Never-screened	1,742	84,910.2	20.5	1.00 (reference)	1.00 (reference)	1.00 (reference)	1.00 (reference)
Screened	1,729	158,590.1	10.9	0.53 (0.50, 0.57)	0.52 (0.48, 0.55)	0.67 (0.62, 0.71)	0.68 (0.63, 0.72)

BC death							
Never-screened	1,371	84,910.2	16.1	1.00 (reference)	1.00 (reference)	1.00 (reference)	1.00 (reference)
Screened	1,243	158,590.1	7.8	0.49 (0.45, 0.53)	0.47 (0.44, 0.51)	0.65 (0.60, 0.70)	0.65 (0.60, 0.70)

Non-BC death							
Never-screened	371	84,910.2	4.4	1.00 (reference)	1.00 (reference)	1.00 (reference)	1.00 (reference)
Screened	486	158,590.1	3.1	0.70 (0.61, 0.80)	0.67 (0.59, 0.77)	0.69 (0.60, 0.79)	0.74 (0.64, 0.86)

Values are presented as hazard ratio (95% confidence interval).

BC, breast cancer.

1 Adjusted for age and socioeconomic status.

2 Adjusted for age, socioeconomic status, stage, histological subtype, and anatomic site.

3 Adjusted for age, socioeconomic status, stage, anatomic site, and histological subtype (invasive cancer only, n=21,341).

**Table 3:** HRs for BC death by subgroups according to the screening history

Variables	No. of BC deaths	Person-years	Death rate per 1,000	Fully adjusted HR (95% CI)^1^
Stage at diagnosis				
DCIS				
Never-screened	9	9,417.4	0.96	1.00 (reference)
Screened	14	23,173.5	0.60	0.62 (0.26, 1.43)
Localized				
Never-screened	220	39,763.1	5.53	1.00 (reference)
Screened	266	79,090.9	3.36	0.58 (0.49, 0.70)
Regional				
Never-screened	529	26,923.9	19.65	1.00 (reference)
Screened	551	43,473.4	12.67	0.64 (0.57, 0.73)
Distant				
Never-screened	474	2,796.0	169.53	1.00 (reference)
Screened	297	2,318.0	128.13	0.76 (0.66, 0.89)
Unknown				
Never-screened	139	6,009.9	23.13	1.00 (reference)
Screened	115	10,534.4	10.92	0.48 (0.37, 0.62)

Age at diagnosis (yr)^2^				
40–49				
Never-screened	504	36,728.3	12.11	1.00 (reference)
Screened	406	53,949.7	6.44	0.71 (0.62, 0.81)
50–59				
Never-screened	442	23,675.6	16.74	1.00 (reference)
Screened	454	49,914.5	7.90	0.65 (0.57, 0.74)
60–69				
Never-screened	212	9,933.9	19.78	1.00 (reference)
Screened	252	23,777.7	9.21	0.61 (0.51, 0.74)
≥70				
Never-screened	204	5,155.2	36.05	1.00 (reference)
Screened	117	7,774.7	13.37	0.50 (0.40, 0.63)

HR, hazard ratio; BC, breast cancer; CI, confidence interval; DCIS, ductal carcinoma in situ.

1 Adjusted for age, socioeconomic status, stage, histological subtype, and anatomic site.

2 Adjusted for socioeconomic status, stage, histological subtype, and anatomic site (invasive cancer only).

**Table 4:** HRs for BC death according to time interval and screening frequency

Variables	No. of BC deaths	Person-years	Death rate per 1,000	Fully adjusted HR (95% CI)^1^
Screening frequency (times)				
Never screened	1,362	75,492.9	18.04	1.00 (reference)
1	739	74,426.1	9.93	0.70 (0.64, 0.77)
2	333	38,938.3	8.55	0.62 (0.55, 0.70)
≥3	157	22,052.3	7.12	0.52 (0.44, 0.62)
p-value for trend				<0.001

Time interval since screening (mo)				
Never screened	1,362	75,492.9	18.04	1.00 (reference)
≥36	108	10,498.5	10.29	0.75 (0.62, 0.92)
24–35	111	9,694.3	11.45	0.75 (0.62, 0.91)
12–23	206	22,208.6	9.28	0.65 (0.56, 0.75)
≤11	804	93,015.3	8.64	0.63 (0.57, 0.69)
p-value for trend				<0.001

HR, hazard ratio; BC, breast cancer; CI, confidence interval.

1 Adjusted for age, socioeconomic status, stage, histological subtype, and anatomic site.

## SUPPLEMENTARY MATERIALS

Supplementary Material 1. Long-term survival status of the study cohort according to baseline characteristics and screening history

Supplementary Material 2. Hazard ratios for different causes of death by subgroups according to the screening history

Supplementary Material 3. Hazard ratios for different causes of death by subgroups according to the screening history (Excluding DCIS and distant stage, N=20,307)
