# Supplementary material for: Effect of mammography screening on the long-term survival of breast cancer patients: results from the National Cancer Screening Program in Korea
Source: Epidemiol Health. 2022 Oct 26;44:e2022094. doi: 10.4178/epih.e2022094 (PMC10106549; doi:10.4178/epih.e2022094)
Supplement: Supplementary Material 1. — Long-term survival status of the study cohort according to baseline characteristics and screening history [file epih-44-e2022094-Supplementary-1.docx]

**Supplementary Materials**

Supplementary Material 1. Long-term survival status of the study cohort according to baseline characteristics and screening history

|  | **Long-term survival status** | |  |  |
| --- | --- | --- | --- | --- |
| **Variables** | **Alive** | **Dead** | ***P*-value** | **Hazard ratio**^1^ |
|  | N= 20916 (85.8%) | N=3471 (14.2%) |  | HR (95% CI) |
| **Age at diagnosis (years),** n (%) |  |  |  |  |
| 40–49 | 9337 (89.9) | 1050 (10.1) | <0.001 | 1.00 |
| 50–59 | 7315 (87.0) | 1094 (13.0) |  | 1.31 (1.02–1.43) |
| 60–69 | 3206 (82.0) | 703 (18.0) |  | 1.99 (1.81–2.20) |
| 70–79 | 1058 (62.9) | 624 (37.1) |  | 4.14 (3.74–4.58) |
| **Socioeconomic status,** n (%) |  |  |  |  |
| NHIS, upper 50% | 10897 (88.2) | 1464 (11.8) | <0.001 | 1.00 |
| NHIS, lower 50% | 9327 (84.5) | 1706 (15.5) |  | 1.31 (1.22–1.4) |
| MAP | 692 (69.7) | 301 (30.3) |  | 2.08 (1.83–2.35) |
| **Anatomical site,** n (%) |  |  |  |  |
| Inner part | 3384 (88.3) | 449 (11.7) | <0.001 | 1.00 |
| Outer part | 8491 (83.8) | 1641 (16.2) |  | 1.23 (1.11–1.37) |
| Central portion | 970 (86.1) | 157 (13.9) |  | 1.06 (0.88–1.27) |
| Others | 8071 (86.8) | 1224 (13.2) |  | 1.04 (0.93–1.16) |
| **SEER Stage,** n (%) |  |  |  |  |
| DCIS | 2936 (96.4) | 110 (3.6) | <0.001 | 0.49 (0.4–0.59) |
| Localized | 10510 (92.4) | 864 (7.6) |  | 1.00 |
| Regional | 5879 (81.3) | 1353 (18.7) |  | 2.58 (2.37–2.81) |
| Distant | 232 (22.4) | 802 (77.6) |  | 18.5 (16.75–20.43) |
| Unknown | 1359 (79.9) | 342 (20.1) |  | 2.56 (2.26–2.90) |
| **Histological subtype,** n (%) |  |  |  |  |
| DICS | 2936 (96.4) | 110 (3.6) | <0.001 | 0.48 (0.39–0.58) |
| Ductal carcinoma | 15512 (84.2) | 2920 (15.8) |  | 1.00 |
| Lobular carcinoma | 1010 (87.2) | 148 (12.8) |  | 0.78 (0.66–0.92) |
| Others | 1458 (83.3) | 293 (16.7) |  | 0.95 (0.84–1.08) |
| *HR, hazard ratio; CI, confidence interval; NHIS, National Health Insurance Service; MAP, Medical Aid Program; SEER, Surveillance, Epidemiology, and End Results; DCIS, Ductal carcinoma in situ.*  *^1^ Hazard ratio for all-cause death; adjusted for screening history, age, socioeconomic status, stage, histological subtype, and anatomical site.* | | | | |
