# Supplementary material for: Effect of mammography screening on the long-term survival of breast cancer patients: results from the National Cancer Screening Program in Korea
Source: Epidemiol Health. 2022 Oct 26;44:e2022094. doi: 10.4178/epih.e2022094 (PMC10106549; doi:10.4178/epih.e2022094)
Supplement: Supplementary Material 2. — Hazard ratios for different causes of death by subgroups according to the screening history [file epih-44-e2022094-Supplementary-2.docx]

**Supplementary Materials**

Supplementary Material 2. Hazard ratios for different causes of death by subgroups according to the screening history

|  | **All-cause death**^1^ | **BC death**^1^ | **Non-BC death**^1^ |  |
| --- | --- | --- | --- | --- |
|  | HR (95% CI) | HR (95% CI) | HR (95% CI) |  |
| **Stage at diagnosis** |  |  |  |  |
| **DCIS** |  |  |  |  |
| Never screened | 1.00 | 1.00 | 1.00 |  |
| Screened | 0.43 (0.3–0.63) | 0.62 (0.26–1.43) | 0.39 (0.26–0.60) |  |
| **Localized** |  |  |  |  |
| Never screened | 1.00 | 1.00 | 1.00 |  |
| Screened | 0.68 (0.59–0.78) | 0.58 (0.49–0.70) | 0.83 (0.67–1.02) |  |
| **Regional** |  |  |  |  |
| Never screened | 1.00 | 1.00 | 1.00 |  |
| Screened | 0.64 (0.58–0.72) | 0.64 (0.57–0.73) | 0.64 (0.50–0.80) |  |
| **Distant** |  |  |  |  |
| Never screened | 1.00 | 1.00 | 1.00 |  |
| Screened | 0.77 (0.66–0.89) | 0.76 (0.66–0.89) | 0.88 (0.42–1.84) |  |
| **Unknown** |  |  |  |  |
| Never screened | 1.00 | 1.00 | 1.00 |  |
| Screened | 0.53 (0.43–0.66) | 0.48 (0.37–0.62) | 0.74 (0.48–1.14) |  |
| **Age at diagnosis (years)** | |  |  |  |
| **40**–**49**^2^ |  |  |  |  |
| Never screened | 1.00 | 1.00 | 1.00 |  |
| Screened | 0.72 (0.63–0.81) | 0.71 (0.62–0.81) | 0.75 (0.52–1.08) |  |
| **50**–**59**^2^ |  |  |  |  |
| Never screened | 1.00 | 1.00 | 1.00 |  |
| Screened | 0.69 (0.61–0.78) | 0.65 (0.57–0.74) | 0.97 (0.70–1.33) |  |
| **60**–**69**^2^ |  |  |  |  |
| Never screened | 1.00 | 1.00 | 1.00 |  |
| Screened | 0.64 (0.55–0.75) | 0.61 (0.51–0.74) | 0.71 (0.54–0.94) |  |
| **≥70**^2^ |  |  |  |  |
| Never screened | 1.00 | 1.00 | 1.00 |  |
| Screened | 0.56 (0.47–0.66) | 0.50 (0.40–0.63) | 0.64 (0.50–0.82) |  |
| *HR, hazard ratio; CI, confidence interval; DCIS, ductal carcinoma in situ.*  *^1^Adjusted for age, socioeconomic status, stage, histological subtype, and anatomic site.*  *^2^Adjusted for socioeconomic status, stage, histological subtype, and anatomic site (invasive cancer only).* | | | | |
