# Supplementary material for: Effect of mammography screening on the long-term survival of breast cancer patients: results from the National Cancer Screening Program in Korea
Source: Epidemiol Health. 2022 Oct 26;44:e2022094. doi: 10.4178/epih.e2022094 (PMC10106549; doi:10.4178/epih.e2022094)
Supplement: Supplementary Material 3. — Hazard ratios for different causes of death by subgroups according to the screening history (Excluding DCIS and distant stage, N=20,307) [file epih-44-e2022094-Supplementary-3.docx]

**Supplementary Materials**

Supplementary Material 3. Hazard ratios for different causes of death by subgroups according to the screening history (Excluding DCIS and distant stage, N=20,307)

|  | **All-cause death**^1^ | **BC death**^1^ | **Non-BC death**^1^ |
| --- | --- | --- | --- |
|  | HR (95% CI) | HR (95% CI) | HR (95% CI) |
| **Overall** |  |  |  |
| Never screened | 1.00 | 1.00 | 1.00 |
| Screened | 0.64 (0.6–0.7) | 0.61 (0.55–0.66) | 0.74 (0.64–0.85) |
| **Age at diagnosis (years)** | |  |  |
| **Age 40**–**49** |  |  |  |
| Never screened | 1.00 | 1.00 | 1.00 |
| Screened | 0.71 (0.61–0.82) | 0.70 (0.60–0.82) | 0.77 (0.53–1.12) |
| **Age 50**–**59** |  |  |  |
| Never screened | 1.00 | 1.00 | 1.00 |
| Screened | 0.64 (0.56–0.74) | 0.59 (0.5–0.69) | 0.91 (0.66–1.26) |
| **Age 60**–**69** |  |  |  |
| Never screened | 1.00 | 1.00 | 1.00 |
| Screened | 0.64 (0.54–0.76) | 0.59 (0.48–0.74) | 0.71 (0.54–0.95) |
| **Age ≥70** |  |  |  |
| Never screened | 1.00 | 1.00 | 1.00 |
| Screened | 0.53 (0.45–0.63) | 0.44 (0.34–0.56) | 0.65 (0.5–0.83) |
| *HR, hazard ratio; CI, confidence interval.*  *^1^Adjusted for age, socioeconomic status, stage, histological subtype, and anatomic site.* | | | |
